# Scaling-up and scaling-out the Systems Analysis and Improvement Approach to optimize the hypertension diagnosis and care cascade for HIV infected individuals (SCALE SAIA-HTN): a stepped-wedge cluster randomized trial

**DOI:** 10.1186/s43058-024-00564-1

**Published:** 2024-03-20

**Authors:** Carmen E. Hazim, Igor Dobe, Stephen Pope, Kristjana H. Ásbjörnsdóttir, Orvalho Augusto, Fernando Pereira Bruno, Sergio Chicumbe, Norberto Lumbandali, Inocêncio Mate, Elso Ofumhan, Sam Patel, Riaze Rafik, Kenneth Sherr, Veronica Tonwe, Onei Uetela, David Watkins, Sarah Gimbel, Ana O. Mocumbi

**Affiliations:** 1 Department of Global Health, University of Washington, Seattle, WA USA; 2 Department of Child, Family, and Population Health Nursing, University of Washington, Seattle, WA USA; 3 Instituto Nacional de Saúde, Vila de Marracuene, Província de Maputo Mozambique; 4 Centre of Public Health Sciences, University of Iceland, Reykjavík, Iceland; 5 Department of Epidemiology, University of Washington, Seattle, WA USA; 6 Faculty of Medicine, Universidade Eduardo Mondlane, Maputo, Mozambique; 7 Center for Translation Research and Implementation Science, National Heart, Lung, and Blood Institute, National Institutes of Health, Bethesda, Washington D.C USA; 8 Mozambique Institute for Health Education and Research, Maputo, Mozambique; 9 Department of Industrial & Systems Engineering, University of Washington, Seattle, WA USA; 10 Division of General Internal Medicine, Harborview Medical Center, Seattle, WA USA

**Keywords:** Systems analysis and improvement approach (SAIA), Hypertension, HIV, Service integration, RE-AIM, CFIR, Process mapping, Cascade analysis, Continuous quality improvement, Stepped wedge

## Abstract

**Background:**

Undiagnosed and untreated hypertension is a main driver of cardiovascular disease and disproportionately affects persons living with HIV (PLHIV) in low- and middle-income countries. Across sub-Saharan Africa, guideline application to screen and manage hypertension among PLHIV is inconsistent due to poor service readiness, low health worker motivation, and limited integration of hypertension screening and management within HIV care services. In Mozambique, where the adult HIV prevalence is over 13%, an estimated 39% of adults have hypertension. As the only scaled chronic care service in the county, the HIV treatment platform presents an opportunity to standardize and scale hypertension care services.

Low-cost, multi-component systems-level strategies such as the Systems Analysis and Improvement Approach (SAIA) have been found effective at integrating hypertension and HIV services to improve the effectiveness of hypertension care delivery for PLHIV, reduce drop-offs in care, and improve service quality. To build off lessons learned from a recently completed cluster randomized trial (SAIA-HTN) and establish a robust evidence base on the effectiveness of SAIA at scale, we evaluated a scaled-delivery model of SAIA (SCALE SAIA-HTN) using existing district health management structures to facilitate SAIA across six districts of Maputo Province, Mozambique.

**Methods:**

This study employs a stepped-wedge design with randomization at the district level. The SAIA strategy will be “scaled up” with delivery by district health supervisors (rather than research staff) and will be “scaled out” via expansion to Southern Mozambique, to 18 facilities across six districts in Maputo Province. SCALE SAIA-HTN will be introduced over three, 9-month waves of intensive intervention, where technical support will be provided to facilities and district managers by study team members from the Mozambican National Institute of Health. Our evaluation of SCALE SAIA-HTN will be guided by the RE-AIM framework and will seek to estimate the budget impact from the payer’s perspective.

**Discussion:**

SAIA packages user-friendly systems engineering tools to support decision-making by frontline health workers and to identify low-cost, contextually relevant improvement strategies. By integrating SAIA delivery into routine management structures, this pragmatic trial will determine an effective strategy for national scale-up and inform program planning.

**Trial registration:**

ClinicalTrials.gov NCT05002322 (registered 02/15/2023).

Contributions to the literature

- Our study examines whether the delivery of hypertension care and related health outcomes for people living with HIV are improved following introduction of a user-friendly, low-cost package of systems engineering tools delivered iteratively at scale by Ministry of Health district health supervisors to public sector health facility teams in a low-income country.
- Research on systems engineering tools within healthcare settings has largely come from high-resourced health systems in high-income countries, with minimal experiences and lessons learned from low-income countries.
- This work provides targeted guidance on how to scale effective implementation strategies through routine management structures, which has been underreported in previous implementation trials.

## Background

Undiagnosed and untreated hypertension is one of the largest drivers of cardiovascular disease (CVD) in sub-Saharan Africa [1–4], with a reported hypertension prevalence of 29% [5]. Globally, approximately 9 million individuals infected with the human immunodeficiency virus (HIV) have hypertension, with 59% of the burden in sub-Saharan Africa [6]. Comorbidity of hypertension and HIV is increasingly common due to an aging HIV-infected population and the expansion of antiretroviral therapy (ART), which is a risk factor for hypertension [7]. Improvements in ART access and effectiveness have led to increased survivorship and thus increases in the number of older people living with HIV (PLHIV) and comorbid hypertension [8–12]. As a result, hypertension is more prevalent among PLHIV [7, 13].

In Mozambique, where the prevalence of HIV is over 13% [14], an estimated 39% of adults (25–64 years old) have hypertension [15]. Compared to other countries in sub-Saharan Africa, Mozambique has one of the lowest proportion of adults (14.5%) who are aware of their hypertension status, and of these, only half are accessing treatment [15]. Furthermore, only 3% of the total adult population with hypertension in Mozambique have their condition controlled. A recent call to action by the World Hypertension League has pushed for improved hypertension management across Africa with the goals of 80% of adults with hypertension to be diagnosed, 80% of those diagnosed to be on treatment, and 80% of those treated to have controlled hypertension [16].

The hypertension cascade for PLHIV includes screening for hypertension in outpatient services, hypertension diagnosis, prescription of hypertension management medications to eligible patients, medication pick up, and finally controlled hypertension [17]. However, multi-level barriers at the individual (patient and/or caregiver) [18, 19], interpersonal (provider) [20, 21], health systems [22], and policy levels prevent optimal cascade implementation [23]. While the Pan-African Society of Cardiology (PASCAR) recommends routine hypertension screening and management of all adult PLHIV [24], guideline application is low and inconsistent due to poor service readiness, low health worker motivation, lack of performance accountability, and limited integration of hypertension screening and management within HIV care services [8]. In addition, low access to care and health system inefficiencies are important factors that limit hypertension care effectiveness for PLHIV, while weaknesses in data management lead to the underutilization of data for optimization of HIV outpatient services [25–28].

Systems engineering tools have been increasingly applied to identify drivers of health system inefficiencies, support provider decision-making to prioritize solutions, and improve integration of multi-step health service cascades (such as the hypertension cascade for PLHIV) using simple, low-cost, iterative adaptations in service delivery processes [29–31]. The Systems Analysis and Improvement Approach (SAIA) is an evidence-based implementation strategy that bundles systems engineering tools into a five-step, facility-level package to provide clinic staff and managers a system-wide view of their cascade performance, identify priority areas for improvement, discern modifiable opportunities for improvement, and test workflow modifications [32–34]. The process is iterative, which means care teams can continue to use the SAIA approach to further improve care and respond to new bottlenecks that arise. During initial testing, SAIA was applied to optimize the prevention of mother-to-child transmission of HIV in three sub-Saharan African countries and demonstrated dramatic cascade improvements, as well as high penetration, acceptability, and feasible integration into routine service management activities [33, 34]. SAIA has been adapted and applied to multiple care cascades in the USA and multiple countries in sub-Saharan Africa including but not limited to pediatric HIV testing [35], HIV testing in family planning services [36], cervical cancer screening and prevention [37], severe mental illness diagnosis and management [38], malaria diagnosis and treatment, and Naloxone distribution [39].

SAIA’s application to the hypertension cascade among PLHIV was assessed in a recent cluster randomized trial in central Mozambique (SAIA-HTN: R01HL142412, NCT04088656). The trial demonstrated cascade gains and the strategy was found appropriate by users when applied to the hypertension cascade within HIV care settings [17, 40, 41]. As integration of hypertension screening, diagnosis, and management services for PLHIV in low- and middle-income countries (LMICs) requires strategies that are scalable (reach a high proportion of the target population across outpatient HIV systems), affordable (require modest resources to align with current health sector investments), and sustainable (integrated into and led by public sector health systems), while still being effective, additional evidence is needed on implementation and costs of the SAIA-HTN model at scale.

### Goals and objectives

The overall goal of this study is to develop and evaluate a scaled delivery model for SAIA-HTN (SCALE SAIA-HTN) to inform national scale-up. To do this, we will conduct a three-wave, stepped wedge cluster randomized trial in which districts are the unit of randomization. The strategy (SAIA-HTN) will be “scaled up” via delivery by district health supervisors (rather than research staff) and will be “scaled out” via expansion [42] to 18 facilities across six districts in Maputo Province, in southern Mozambique. SCALE SAIA-HTN’s specific objectives are to:

1. Develop a district-based dissemination and implementation strategy for SCALE SAIA-HTN using the RE-AIM model [43] to evaluate the programs’ Reach, Effectiveness, Adoption, Implementation, and Maintenance
2. Determine the costs of SCALE SAIA-HTN for care cascade optimization, including total and incremental costs of integrating hypertension diagnosis and management into HIV care.

### Study rationale

Our study design has several advantages. It is robust (with high power) to detect changes in service-level process and individual-level clinical outcomes, attribute change to our implementation strategy, and address time trends and has the bias control benefits of randomized designs. The phased-in design is logistically feasible, allowing us to reach a larger number of districts and facilities than a parallel cluster trial. Ethically, it does not require withholding an intervention that is known to improve services. Finally, it provides an opportunity to analyze maturation patterns in implementation strategy effect and maintenance over time, including during the intensive and sustainment phases.

## Methods

### Overview of the SAIA implementation strategy

**Fig. 1:**
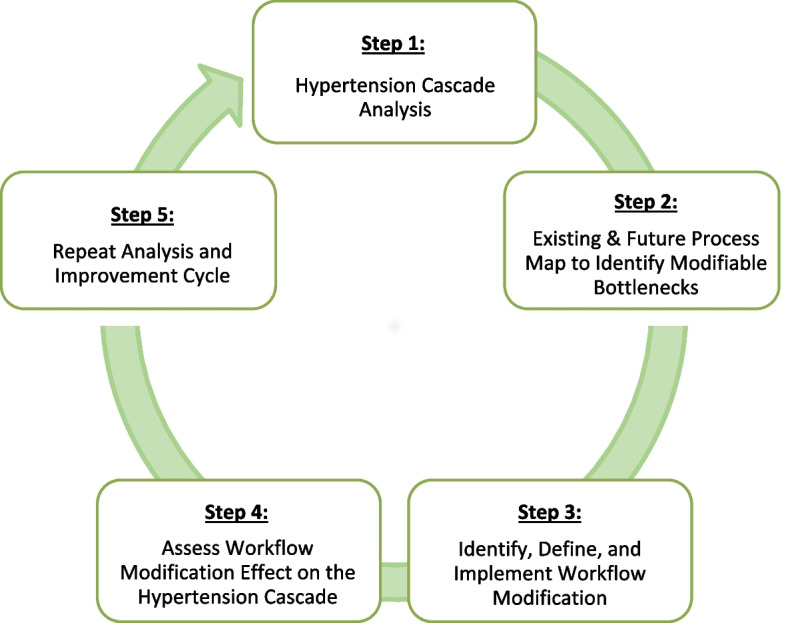
Systems Analysis and Improvement Approach (SAIA) implementation strategy

The core components of the SAIA implementation strategy [32] and its adaptation to the hypertension cascade for PLHIV [17] have been previously described. In brief, the SAIA implementation strategy is an iterative, cyclical process (Fig. 1) applied and repeated at the facility level monthly. Tailored to the hypertension care cascade, SAIA-HTN is a strategy that engages health care workers in the use of a series of systems engineering tools including cascade analysis using the Hypertension Cascade Analysis Tool (HCAT), process mapping, and continuous quality improvement (CQI), in order to improve and sustain iterative improvement to their service delivery [31]. As part of each “SAIA cycle,” the HCAT is first completed using routine data to quantify patient flow through the hypertension care cascade, providing clinic staff and managers with a systems view of cascade performance [44–46]. This is followed by process mapping of the steps patients take at their facility to obtain care, which helps staff come to a consensus on how their system operates, supports the identification of modifiable bottlenecks, and guides discussion and group problem-solving on opportunities for workflow modifications [47]. Together, HCAT and process mapping tools assist care teams to identify redundancies and inefficiencies and to prioritize opportunities for process improvement which in turn supports CQI activities. CQI enables care teams to prioritize areas for improvement each month, discern modifiable solutions, create an action plan, and test and reflect on workflow modifications or “micro-interventions”.

### SCALE SAIA-HTN trial design

**Fig. 2:**
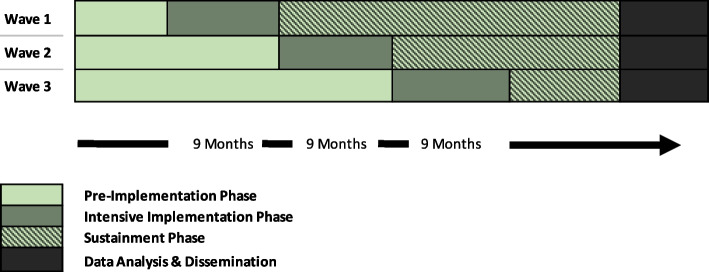
SCALE SAIA-HTN stepped wedge implementation timeline

The SCALE SAIA-HTN trial will employ a stepped wedge design to randomly allocate six districts in Maputo Province, Mozambique, into three implementation waves, each staggered by 9 months (Figure 2). In line with other trials of SAIA at scale, districts were selected as the unit of randomization because district management structures have oversight, responsibility, and authority for health services within their districts; can access resources to address health facility needs; and can establish processes and management protocols within subordinate health facilities.

The trial will begin with a pre-implementation phase in which an electronic individual-level (patient) data monitoring system will be introduced. Each wave will start with a 9-month intensive phase, during which district health supervisors will deliver SAIA for hypertension optimization in three enrolled health facilities in each of their respective districts (18 facilities total). During the intensive phase, the district supervisors will be accompanied and mentored by research staff from the Mozambican National Institute of Health (Instituto Nacional de Saúde, INS) and the Universidade Eduardo Mondlane (UEM). Subsequently, and depending on wave allocation, a 9-, 18-, or 27-month sustainment phase will take place with implementation led independently by district supervisors. During the sustainment phase, to approximate real-world conditions in the roll-out, district supervisors will be funded for travel to enrolled facilities and provided with phone and Internet credit but will not receive hands-on supervision and mentorship from research staff.

### Study setting and population

**Fig. 3:**
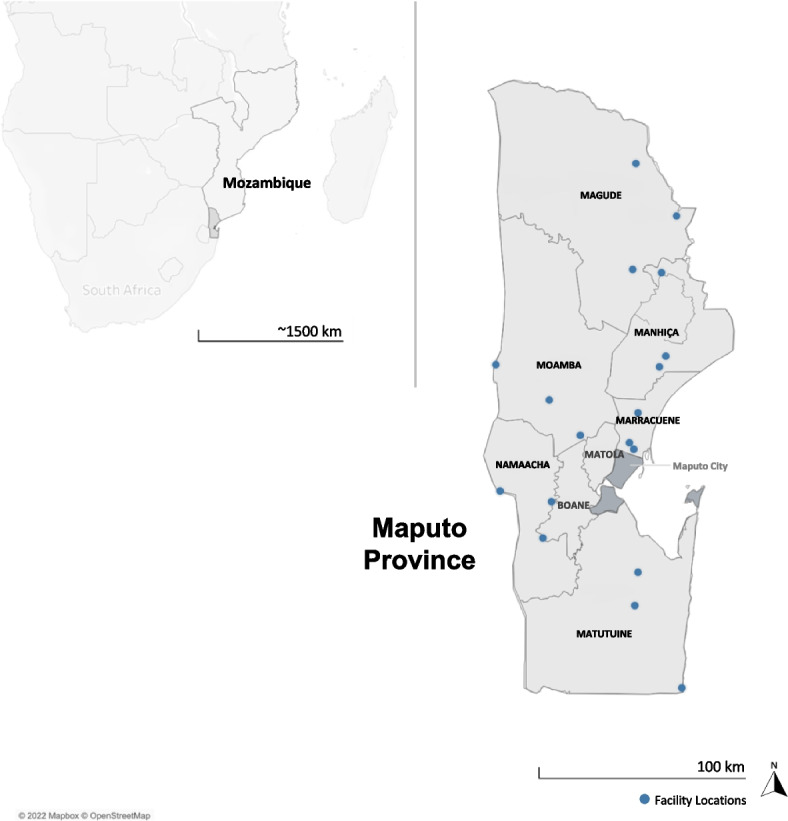
Map of Maputo Province, Mozambique, and locations of 18 enrolled facilities. Enrolled facility locations, represented by blue dots (*n* = 18), are spread across six of the eight districts in Maputo Province, Mozambique

Maputo Province (Figure 3), located in southern Mozambique, has a population of approximately two million inhabitants and a higher adult HIV prevalence than the national average (23%, compared to 13% nationally) [48, 49]. Across its eight districts, Maputo Province has 112 health facilities, including 11 health posts, 97 health centers, three district hospitals, and one tertiary provincial hospital [50]. Approximately 79% of these facilities have the equipment and skills needed to measure, diagnose, monitor, and manage hypertension. Across the country, over 98% of formal health services are offered through public sector clinics [51], and primary care service utilization is high [52].

### Enrollment criteria and randomization

Across Maputo Province, six districts have been included in the SCALE SAIA-HTN trial, providing a robust understanding of implementation challenges and opportunities and strategy effectiveness in this setting. Using a random number generator, districts have been randomly allocated without restriction to implementation wave one, two, or three, with two districts allocated to each wave. To maximize the potential impact of SCALE SAIA-HTN, the three highest HIV-patient volume health facilities (i.e., facilities with the greatest number of active PLHIV patients on ART) in each enrolled district of Maputo Province have been selected for inclusion in the study (Table 1). All three selected health facilities within the same district have been allocated to the same wave. Delivery of SAIA-HTN occurs with the healthcare team at the facility level. Patients will not be directly enrolled in the study. However, to assess the impact of SAIA on patient-level outcomes, de-identified data on all adult PLHIV (18+ years old) with hypertension, regardless of established or novel hypertension diagnosis, will be captured by the study if they access outpatient services at enrolled facilities during the trial.

**Table 1:** SCALE SAIA-HTN enrolled districts and facilities

**Maputo Province**			
	**District**	**Health facility**	**Patients on ART**
**Wave 1**	Magude	Magude	5206
		Chichuco	309
		Motaze	822
	Matutuíne	Matutuíne	1674
		Ponta de Ouro	1584
		Salamanga	601
**Wave 2**	Namaacha	Namaacha	2296
		Mahelane	568
		Mafuiane	999
	Moamba	Moamba	4345
		Ressano Garcia	2606
		Tenga	2029
**Wave 3**	Manhiça	Manhiça	10,397
		Xinavane	5758
		Maragra	2080
	Marracuene	Nhongonhane	3555
		Habel Jafar	2487
		Ricatla	1590
**Total**	6	18	48,906

### Process for introducing SAIA

The SCALE SAIA-HTN trial will use the foundational infrastructure developed for the original SAIA-HTN trial including the HCAT, implementation guides and tools, and data source mapping [17]. In the pre-implementation phase, the delivery schedule, standard operating procedures (SOPs), and training materials from the original trial will be adapted and refined. For the collection of relevant individual-level, service-level, and implementation metrics, an electronic record management system will be established using CommCare, an open-source and HIPAA-compliant mobile platform by Dimagi, Inc. [53]. CommCare will be used to (1) capture relevant study outcomes from Ministry of Health outpatient HIV registries and hypertension-specific patient charts, (2) complete readiness surveys and HCATs, and (3) document developed process maps and quality improvement action plans. Data clerks will be trained in data collection procedures and a pilot of the CommCare platform and study tools will be conducted.

At the start of each *intensive* wave, the SCALE SAIA-HTN study team will provide district health supervisors and health facility staff with a supply of sphygmomanometers, blood pressure cuffs, and batteries, and a 3-day training on the SAIA implementation strategy. Training will include an introduction to each component of the SAIA methodology, practice sessions with the SAIA HCAT, process mapping, and action planning tools (https://www.saia-strategy.com/tools), and a review of the implementation schedule. The first day of the training will be conducted in the district capital with district supervisors and facility managers from the three selected health facilities, while the second and third days of the training will be conducted onsite and tailored to each facility. During the facility-based training days, health facility teams will complete their first SAIA cycle by working with study personnel and district supervisors to populate and interpret the HCAT, develop process maps, define one to two micro-interventions, assign roles, and agree upon measures to monitor these modifications. In the first month of SAIA, facility teams will receive two visits by district supervisors and study personnel, followed by monthly visits for all subsequent SAIA cycles throughout the intensive phase. Through participation in monthly visits during the 9-month intensive phase, study personnel will actively mentor both district supervisors and frontline healthcare teams to support their attainment of adequate SAIA-HTN knowledge and skills, and thus lay the groundwork for the strategy’s subsequent sustainment.

In the *sustainment phase* (length by wave will be 27, 18, or 9 months, determined by implementation wave allocation), it is expected that district supervisors will independently lead SAIA-HTN implementation monthly with facility teams, with small financial subsidies to support travel to clinics and meeting snacks, but without intensive support and supervision from study personnel. In the case of district supervisor turnover during the intensive or sustainment phases, the study team will train the new supervisor. The provision of flexible facility support will continue throughout both the intensive and sustainment phases of the SCALE SAIA-HTN trial to address basic needs for hypertension clinical management (e.g., blood pressure cuffs, sphygmomanometers), as well as needs arising from proposed workflow modifications.

### SCALE SAIA-HTN impact evaluation: application of the RE-AIM framework

**Fig. 4:**
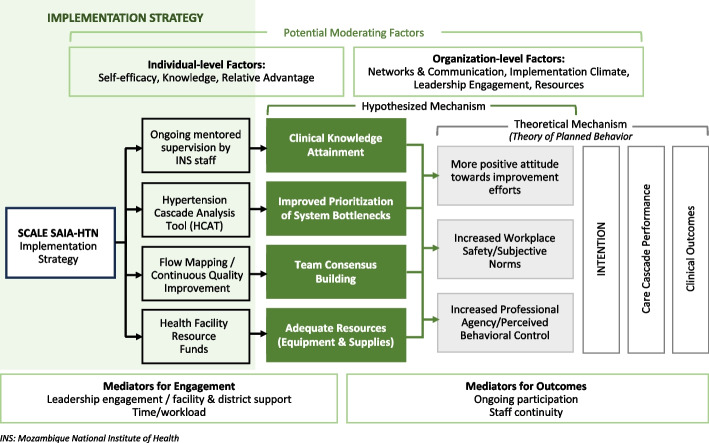
SCALE SAIA-HTN conceptual framework. INS, National Institute of Health

We will use a mixed-methods evaluation guided by the RE-AIM framework to evaluate implementation outcomes, service-level process outcomes, and individual-level clinical outcomes of the district-led SCALE SAIA-HTN strategy. RE-AIM is commonly used to assess public health impacts of complex interventions across individual, organizational, and policy levels [43]. Data collection for service-level processes and individual-level clinical outcomes will be continuous, with implementation outcome measures collected at monthly intervals or at set times such as the beginning or end of the intensive phase of implementation. Our evaluation uses a theory of change approach guided by a conceptual framework describing SCALE SAIA-HTN strategy components, mechanisms for effect, implementation mediators/moderators, and theoretical underpinnings (Figure 4). Incorporating established implementation science methods into our assessment of SCALE SAIA-HTN will provide a comprehensive understanding of the components that determine the strategy’s public health impact and will generate actionable insights to inform planning for post-trial expansion.

*Exposure definition:* Enrolled facilities will be considered “unexposed” prior to the initiation of SCALE SAIA-HTN in their districts and thereafter will be considered “exposed.” Individuals (adult PLHIV with hypertension) will be considered “exposed” if they access care at an enrolled facility that is considered exposed to SCALE SAIA-HTN.

#### Reach

Reach, along with intervention efficacy, is an active ingredient in effectiveness and population health impact*.* As our strategy is delivered at the facility level, we will use study reports to estimate the proportion of all facilities in the six enrolled districts within Maputo province reached by or exposed to SCALE SAIA-HTN (target: 27%, or 18 of 67 facilities in enrolled districts). Moreover, we will use routine administrative health data to estimate the number and proportion of adult PLHIV with hypertension reached (i.e., exposed) across the enrolled districts (the number of PLHIV with hypertension attending outpatient services in enrolled facilities/total number of estimated eligible PLHIV adults with hypertension; target: 80%). If adopted and implemented as intended, we expect the number of adult PLHIV with hypertension who are not reached by SCALE SAIA-HTN to be low given that we are selecting the highest volume facilities in each district. This analysis will help to identify sub-groups of adult PLHIV with hypertension whose needs are not met, including those who attend facilities not covered by SCALE SAIA-HTN, or those who do not seek services.

#### Effectiveness

To assess the effectiveness of SCALE SAIA-HTN on the hypertension cascade for PLHIV, we will measure individual-level clinical outcomes of hypertension treatment effectiveness and service-level process outcomes that evaluate improved flow across the hypertension cascade (Table 2). The primary individual-level clinical effectiveness outcome will be controlled hypertension at any time after treatment initiation. Secondary individual-level outcomes will include controlled hypertension at 3 months after treatment initiation and HIV viral load suppression. The service-level process outcomes will mirror the hypertension cascade and include hypertension screening (i.e., proportion of PLHIV screened for hypertension in outpatient services), hypertension diagnosis (i.e., proportion of PLHIV screened for hypertension in outpatient services who receive or have previously received a hypertension diagnosis and are thus eligible for hypertension medications), hypertension treatment initiation (i.e., proportion of eligible PLHIV prescribed anti-hypertensive medications), hypertension medication pick up, and proportion of patients achieving > 90% adherence to hypertension treatment as assessed by medication possession ratios at 3 and 6 months (all reflecting Ministry of Health guidelines).

**Table 2:** SCALE SAIA-HTN effectiveness outcome measures

Measure	Definition	Source
Individual clinical outcomes		
Controlled hypertension^a^	% PLHIV, previously diagnosed hypertension, with controlled hypertension after treatment start	Hypertension Patient Form
HIV viral load suppression	% PLHIV on hypertension meds with viral load suppressed (< 20 copies/mL)	Viral Load Register within Outpatient Register
Service-level process outcomes		
Hypertension screening	% PLHIV at outpatient consults with blood pressure screening for hypertension per month	Outpatient Register
Hypertension diagnosis	% PLHIV identified hypertensive in outpatient consults, per month	Hypertension Patient Form
Treatment initiation	% eligible PLHIV-prescribed hypertension meds, per month	Hypertension Patient Form
Hypertension medication pick up	% PLHIV at a return visit prescribed hypertension meds, who picked them up at the previous visit	Hypertension Patient Form
Hypertension medication adherence	% PLHIV who completed a visit on time or < 10% late resulting in medication possession ratio of >90%	Hypertension Patient Form

^a^Primary outcome of interest

All outcome measures will be binary upon collection. Service-level process outcomes will be based on monthly aggregates, with the eligible PLHIV patients presenting at each point in the hypertension care cascade as denominators. Each patient’s progression through a given step will be defined as successful or unsuccessful. Progression through earlier steps will not be a prerequisite; patients will remain eligible for later steps in the cascade despite failing to complete earlier steps.

*Data sources:* The general outpatient HIV registry located in each outpatient consultation room, complemented by the hypertension patient forms, facilitates longitudinal tracking of hypertension utilization and outcomes for PLHIV. As part of routine care, each PLHIV diagnosed is assigned a unique identification number in the form of a QR code that links across service points and clinics. This identification number will be used to abstract registry data for study outcomes. Data from the registry and patient charts will be abstracted and entered into the CommCare mobile data collection platform daily by SAIA data clerks in each facility [53]. Data quality audits will be carried out at randomly selected clinics each year and results used in sensitivity analyses to quantify the potential impact of errors on effectiveness measures.

*Power and sample size:* Preliminary data from the study facilities in September–December 2022 show a mean of 32 “second visits” (return visits after hypertension treatment initiation) per month per facility. District totals across all three facilities for the 3-month period ranged from 140 in the Matutuine district to 199 in the Marracuene district. While a subset of patients’ returns is after 3 months under differentiated care models, the majority of return visits are scheduled for 6 months; thus, we conservatively estimate that a 9-month wave will allow us to observe 3 months’ worth of return visits among patients who were exposed to SAIA-HTN at their first visit, for a minimum of 140 return visits. Among return visits, the mean proportion with controlled hypertension was 23.4%. For the primary individual-level clinical outcome of controlled hypertension at a return visit, assuming a baseline of 23.4%, with three waves of two districts each, 140 return visits per wave per district, and assuming an intra-cluster correlation of 0.2 and *α* = 0.05, we will have 80% power to detect an absolute increase of 6.9 to 30.3%.

*Data analysis:* Service-level outcome data will be analyzed using mixed-effects logistic regression, with clustering by district. Difference in means of service-level process outcomes (Table 2) by exposure status will be reported. Individual-level clinical outcomes will be analyzed using odds ratios estimated by mixed-effects logistic regression for binary outcomes, with two levels of clustering by facility and district.

In each district, we will consider SCALE SAIA-HTN’s effect to be fixed throughout the exposed period. Effect modification of the SCALE SAIA-HTN effect by facility-level factors such as patient volume, provider training, and distance from the district office will be assessed using interaction terms, with potential effect modifiers assessed categorically or dichotomized depending on distribution. Potential adjustment variables to be assessed for inclusion in the models include calendar year and individual-level factors such as age, body mass index, timing of HIV diagnosis, and comorbid conditions. Multivariable models including potential adjustment variables will be run and model fit compared using AIC, with only those that improve model fit retained. Sensitivity analyses will (a) test for a time trend in SCALE SAIA-HTN’s effectiveness using an interaction term between SCALE SAIA-HTN’s status and months since roll-out and (b) assess the potential impact of mis-entered data.

#### Adoption

For the purposes of our study, adoption will be defined as the proportion of enrolled districts and facilities attending training and initiating at least one SAIA cycle. Based on previous SAIA research [54], we expect high acceptance of SCALE SAIA-HTN (target = 95% adoption). To describe the determinants of adoption, we will assess both structural and organizational readiness. Structural readiness will be assessed at all study facilities at the beginning and end of each intensive implementation wave to quantify facility structural determinants of implementation of hypertension management guidelines. Assessments will use a previously developed and refined questionnaire adapted from the World Health Organization (WHO) Service Availability and Readiness Assessment [55] and Demographic and Health Survey Service Provision Assessment [56].

Administration of the Organizational Readiness for Implementation Change (ORIC) [57] scale, our assessment of organizational readiness, will take place within the first 3 months of each implementation wave and target six health managers and frontline staff working across the HIV-hypertension cascade and facility leadership in each clinic (*n* = 108). We will use the ORIC to determine the extent to which organizational members are psychologically and behaviorally prepared to implement organizational change [58]. This 12-item Likert-type scale is broken into the domains of change commitment, the shared resolve to implement a change (4 items), and change efficacy, the shared belief of the collective capacity to implement change (8 items). ORIC has demonstrated reliability, content validity, structural validity, structural invariance, and known-groups validity in field application [57].

Our analyses will test whether there is sufficient inter-rater reliability and inter-rater agreement to aggregate individual responses to the facility level [59–62]. If findings do not justify aggregation (and for the district cases), a measure of intra-facility and intra-district variability in readiness will be used in the analysis rather than a facility-level mean [60, 62]. The resulting analysis will provide readiness profiles for each district and facility as they initiate implementation, which will complement data on reach, implementation, and effectiveness.

#### Implementation

Implementation will be assessed at the facility, district, and provincial levels. We will employ the Consolidated Framework for Implementation Research (CFIR), a determinant-based framework, to (1) guide an assessment of implementation processes and fidelity, (2) describe core elements of SCALE SAIA-HTN implementation, and (3) identify the drivers of success and failure across implementing health facilities.

Implementation processes and fidelity will be tracked quantitatively using CommCare and will include the number and frequency of SAIA cycles, the number of micro-interventions tested, and the content and results of the micro-interventions in each action plan. Implementation fidelity by facility-month will be defined as the occurrence of a monthly SAIA meeting, use of the HCAT, and development of or revision of an action plan. We will use the fidelity measure to identify facilities of high and low performance for a subsequent qualitative evaluation of determinants of successful implementation.

Core elements and determinants of implementation success or failure will be assessed qualitatively via in-depth interviews (IDIs) and focus group discussions (FGDs) with facility, district, and provincial staff. Data collection will use available CFIR tools (http://cfirguide.org) adapted to focus on select constructs from each CFIR domain (Table 3). FGDs and IDIs will be carried out after each wave has transitioned from the intensive to sustainment phase of implementation, as previous SAIA research has found insufficient heterogeneity of implementation fidelity under the intensive support condition to assess drivers of differential implementation [54]. A total of 18 FGDs (one per facility) will be conducted in the 3 months following the end of each intensive phase (i.e., during the sustainment phase) and will consist of seven to 10 participants to encourage conversation without being overwhelming or intimidating in size [63] At the end of the study (i.e., 9 months after the third wave districts have completed their intensive phase), two additional FGDs will be held, one with representatives from two high-performing health facilities (measured as consistent engagement and fidelity to the SCALE SAIA-HTN strategy) and one with representatives from two low-performing health facilities across the entire study. FGDs will seek to capture the shared experience of those receiving SAIA-HTN as it adapts to a scaled district model.

**Table 3:** CFIR data collection

**I. Intervention characteristics**	
Intervention source	1
Evidence strength and quality	1
Relative advantage	1
Adaptability	^a^
Trialability	^a^
Complexity	1
Design quality and package	1
Cost	1
**II. Outer setting**	
Patient needs and resources	^a^
Cosmopolitanism	^a^
Peer pressure	1
External policy and incentives	1
**III. Inner setting**	
Structural characteristics	2
Networks and communications	1
Culture	1
Implementation climate	1
Readiness for implementation	1
**IV. Characteristics of individuals**	
Knowledge and beliefs about intervention	1
Self-efficacy	1
Individual stage of change	1
Individual identification with organization	1
Other personal attributes	1
**V. Process**	
Planning	^a^
Engaging	1
Executing	2
Reflection and evaluation	1

1 = qualitative data, 2 = quantitative data

^a^No primary data collection planned

In addition to the FGDs**,** 64 semi-structured IDIs will be conducted to highlight the individual experiences with disseminating and implementing SCALE SAIA-HTN and capture adaptations and changes over time, such as staff attitudes or identification with the organization. As each district typically has two district supervisors, an IDI with each district supervisor (for HIV and non-communicable diseases) will take place at the end of each respective intensive phase (four per wave) and again at the end of the study. IDIs will also be conducted at the end of each respective intensive phase with one to two health facility managers from each health facility (6 per wave). At the endpoint of the study, to complement the FGDs with high- and low-performing facilities and uncover salient determinants of implementation, IDIs will be held with managers from the two highest- and two lowest-performing facilities. Lastly, at the mid-point and end-line of the study, IDIs will be conducted with two provincial managers (HIV and non-communicable diseases) for Maputo Province. Recruitment will prioritize a balance of participant representation across service provider roles and gender.

FGDs and IDIs will be conducted in Portuguese by an experienced facilitator and accompanying note-taker and will be audio-recorded, transcribed, and translated into English. Two primary coders will independently code transcripts from the IDIs and FGDs and coordinating their coding to create a codebook and conduct thematic analysis. Thematic analysis of the qualitative data will follow CFIR domains and constructs (Table 3), determine core components of SCALE SAIA-HTN, describe adaptations applied by district managers, to distinguish the content and structure of training, materials, and mentorship compared to how it was received and implemented, contextualize implementation processes and structural readiness survey findings, and support an understanding of group norms (via FGDs), and minority opinions (via IDIs) on determinants of implementation.

#### Maintenance

At the organizational level, we will measure the extent to which SCALE SAIA-HTN is institutionalized and sustained over time, including the proportion of districts and facilities that continue to implement SAIA-HTN as designed. The stepped wedge approach facilitates assessing sustainment after the 9-month intensive phase. We will measure the proportion of districts and facilities continuing to implement SCALE SAIA-HTN at 9, 18, and 27 months post-introduction (target = > 90% at 9 months, > 80% at 18 months, and > 65% at 27 months). Continued implementation is defined as holding monthly SAIA-HTN meetings, including the use of the cascade analysis tool, process mapping, and action planning using CQI. We will also probe district and facility perspectives on determinants of sustainment through IDIs and FGDs.

### SCALE SAIA-HTN economic evaluation

We will assess the cost and cost-effectiveness of the program from both societal and healthcare sector perspectives. The latter will include all healthcare costs borne by the Ministry of Health (payer/provider) as well as project costs for implementing SAIA (training, monitoring, etc.) that are currently covered by the SCALE SAIA-HTN project budget but will ultimately be covered by the Ministry of Health in future rollout (Table 4). The comparison scenario in our analysis will be the status quo (i.e., pre-SAIA costs and health outcomes). The time horizon for the analysis will be 10 years following implementation (2025–2034), with discount rates of 3% (base case), 0%, and 6% on both costs and outcomes.

**Table 4:** Economic evaluation summary

**Perspectives**	Societal and healthcare sector. The healthcare sector costs will include payer/provider costs as well as costs currently covered by the SAIA project budget that would be absorbed into the MOH budget at national scale-up.
**Cost estimates**	Costs of SCALE SAIA-HTN delivery, including medical costs relating to screening and treatment; emphasis on measuring the change in costs (time and quantity of resources consumed) after SAIA implementation
**Data collection**	1. SAIA budget reports (project costs) 2. Integrated time-motion and out-of-pocket cost survey (patient time, costs, and % of healthcare visit spent receiving hypertension care) 3. Provider survey (triangulate estimate of % of time spent providing hypertension care) 4. Facility costing tool (prices and context-specific overheads) 5. Patient registry (utilization of hypertension services) 6. Review of government data (e.g., provider salaries, supply chain costs)
**Primary clinical outcomes**	Impact on blood pressure and on the incidence of CVD, including fatal CVD; incremental cost-effectiveness ratios reported as the (1) cost per mmHg blood pressure reduction, (2) cost per patient optimally adherent; (3) cost per CVD death averted, and (4) cost per DALY averted
**Discounting**	3% in the base case; varied from 0 to 6% in sensitivity analysis
**Analytic time frame**	10 years (2025–2034)

#### Measuring costs

Direct project costs will be measured from financial expenditure reports, which will be categorized according to input type and activity. We expect SAIA to increase the time spent providing and receiving hypertension care, as well as the frequency of healthcare visits for hypertension, so our data collection will focus on quantities of time and resources consumed, with prices remaining constant in real terms. Hypertension treatment costs before and after implementation will be measured using a mix of gross costing and micro-costing approaches, and the incremental cost will be calculated as the sum of the direct project costs and the difference in treatment costs after implementation. We will measure quantities and prices before and after implementation by triangulating data from patient registries and from three tools: (1) a patient survey that includes a time-motion component and an out-of-pocket cost component, (2) a provider survey that focuses on the share of overall time spent providing care that can be attributed to hypertension, and (3) a facility costing tool that will measure context-specific overheads and prices of drugs, consumables, and equipment. The patient forms and registries will be abstracted for healthcare utilization data before and after implementation. Additional data that are not available at the facility level will be collected ad hoc from other sources (e.g., average health worker salaries from Ministry of Finance records) or from the literature (e.g., cost of treating acute CVD complications).

#### Cost-effectiveness analysis

The primary effectiveness outcome of controlled hypertension will be the starting point for our cost-effectiveness analysis. A within-study cost-effectiveness analysis will be performed using individual-level data to calculate the cost per mmHg systolic blood pressure reduction and cost per person optimally (≥ 90%) adherent. Additionally, we will develop a state-transition model to project the likelihood of developing new CVD and dying from CVD within the next 10 years; this will be based on patient-level changes in 10-year CVD risk estimated using Framingham-style risk equations or WHO risk charts [64], and we will convert cases and deaths into disability-adjusted life-years (DALYs). We will also do one-way and probabilistic sensitivity analyses and will report our findings in accordance with the Second Panel on Cost-Effectiveness in Health and Medicine [65] and Consolidated Health Economic Evaluation Reporting Standards (CHEERS) checklists [66].

#### Budget impact assessment

Using data on the incremental cost of SCALE SAIA-HTN, we will assess the budget impact for the Ministry of Health to expand and further scale the SAIA implementation strategy in consideration of the eligible population size, and current Ministry of Health budget expenditures [67]. We will follow the guidelines in the ISPOR Task Force on Good Research Practices [68].

## Discussion

SCALE SAIA-HTN is a pragmatic trial to test the scalability of an approach to optimize hypertension diagnosis and management in PLHIV. The implementation approach operates within existing district-level management structures, which is comparable to many other countries with decentralized authority to sub-national jurisdictions closer to where services are offered (e.g., districts, zones, and counties). By providing a practical set of tools that can be integrated into routine functions of district supervisors, the SCALE SAIA-HTN model may facilitate improved health authority management of their facility networks.

Significant resources over the last 20 years have been invested in the development of HIV services in sub-Saharan Africa, making it the first broadly implemented chronic care service platform in many LMICs [69]. The resulting improved health system capacity for chronic care provision presents an opportunity to standardize, integrate, and thus scale additional non-communicable disease interventions, including hypertension screening and management. Systems-level implementation strategies such as SAIA-HTN can support the integration of hypertension care delivery for PLHIV to improve its effectiveness, reduce drop-offs along the hypertension cascade of care, improve service quality, and maximize the availability of efficacious hypertension medicines [44]. SAIA’s approach aligns with research which has shown that the participation of frontline staff and senior management champions such as district health supervisors in quality improvement and optimization processes leads to appropriate, effective, and sustainable solutions; improved health service delivery; and improved clinical outcomes [30, 70, 71]. To ensure the effectiveness of the SCALE SAIA-HTN strategy and continued scale-up, engagement and buy-in from the district non-communicable disease health supervisors delivering SAIA-HTN to facilities in their districts are essential.

## Data Availability

Not applicable.

## Abbreviations

ART: Antiretroviral therapy
CFIR: Consolidated Framework for Implementation Research
CHEERS: Consolidated Health Economic Evaluation Reporting Standards
CQI: Continuous quality improvement
CVD: Cardiovascular disease
DALY: Disability-adjusted life year
FGD: Focus group discussion
HCAT: Hypertension Cascade Analysis Tool
HIV: Human immunodeficiency virus
HTN: Hypertension
IDI: In-depth interview
INS: Instituto Nacional de Saúde (National Institute of Health)
LMIC: Low- and middle-income country
ORIC: Organizational Readiness for Implementing Change
PASCAR: Pan-African Society of Cardiology
PLHIV: People living with HIV
RE-AIM: Reach, Effectiveness, Adoption, Implementation, Maintenance
SAIA: Systems Analysis and Improvement Approach
UEM: Universidade Eduardo Mondlane
WHO: World Health Organization
